# The role of intranasal esketamine in treatment-resistant depression

**DOI:** 10.1192/j.eurpsy.2021.1278

**Published:** 2021-08-13

**Authors:** A. Fraga, D. Esteves-Sousa, J. Facucho-Oliveira, M. Albuquerque, M. Costa, P. Espada-Santos, A. Moutinho

**Affiliations:** Mental Health, Hospital de Cascais, Alcabideche, Portugal

**Keywords:** esketamine, Ketamine, treatment-resistant depression

## Abstract

**Introduction:**

Major depressive disorder (MDD) is a highly prevalent clinical condition with a leading cause of disability worldwide. Unfortunately, about 1/3 of patients with MDD fail to achieve remission despite treatment with multiple antidepressants and are considered to have treatment-resistant depression (TRD). Research showed abnormalities in glutamatergic transmission in neural circuits and antidepressant efficacy with the N-methyl-D-aspartate (NMDA) receptor antagonist, ketamine.

**Objectives:**

The authors elaborate a narrative literature review on the intranasal esketamine as a new-class antidepressant.

**Methods:**

PubMed database searched using the terms “treatment-resistant depression” and “esketamine”.

**Results:**

Ketamine, synthetized from PCP, acts as an antagonist of NMDA receptor, reducing Central Nervous System excitability. One limitation of ketamine for treating depression is that requires intravenous administration, reducing its applicability in outpatient settings. Esketamine, the S-enantiomer of ketamine, developed as an intranasal formulation has a higher affinity for the NMDA receptor. The evidence of the rapid antidepressant effect of intranasal esketamine was first made by Lapidus et al, that demonstrated intranasal esketamine ability to reduce depressive symptomatology. However, some recent studies reported significant acute cardiovascular, psychotomimetic and neurological side-effects. Thus, drug formulation, delivery device, insufflation technique, and individual factors seem to contribute importantly to the tolerability and efficacy of the intranasal administration rote.

**Conclusions:**

There is the need to develop novel treatments providing effective, more rapid-acting, and sustained relief of depressive symptoms, especially in patients with TRD. Intranasal esketamine has shown antidepressant effects in patients with TRD but further investigation is required to strongly reinforce this potential and safety.

